# Interaction of* XRCC1* Arg399Gln Polymorphism and Alcohol Consumption Influences Susceptibility of Esophageal Cancer

**DOI:** 10.1155/2016/9495417

**Published:** 2016-02-01

**Authors:** Man Li, Xia Yu, Zhi-yan Zhang, Chun-long Wu, Hai-long Xu

**Affiliations:** ^1^Endoscopic Department, The Harbin Medical University Cancer Hospital, Harbin, Heilongjiang 150081, China; ^2^Department of Radiology, Armed Police Corps Hospital of Heilongjiang, Harbin, Heilongjiang 150010, China; ^3^Department of Radiology, The Second Affiliated Hospital, Harbin Medical University, Harbin, Heilongjiang 150000, China

## Abstract

*Background*. To explore the correlation between the Arg399Gln polymorphism and susceptibility to esophageal cancer in Korean and Han Chinese individuals in Harbin, China, and its potential interaction with alcohol consumption.* Methods*. This prospective study included 203 patients with esophageal squamous cell carcinoma; 88 were of Korean descent and 115 were of Han Chinese descent. A group of healthy controls included 105 participants of Korean descent and 105 of Han Chinese descent. Genotyping of the Arg399Gln locus of* XRCC1* was performed by PCR-RFLP.* Results*. The allelic and genotypic frequencies were not significantly different between individuals with esophageal cancer and controls or between individuals of Korean and Han Chinese descent (*P* > 0.05). However, when individuals with the wild-type Arg/Arg genotype also consumed alcohol, the risk of esophageal cancer was lower (OR = 3.539; 95% CI = 2.039–6.142; *P* < 0.05).* Conclusions*. The* XRCC1* Arg399Gln polymorphism does not appear to be associated with esophageal cancer in individuals of Korean or Han Chinese descent in Harbin, China. However, alcohol consumption may decrease the risk of esophageal cancer in persons with the wild-type genotype.

## 1. Introduction 

Many cancers result from damage to DNA that eventually affects DNA stability and leads to the malignant transformation of cells. DNA damage must be repaired by one of four cellular processes, base excision repair, nucleotide excision repair, mismatch repair, or double-strand break repair, and alterations in the repair process can allow DNA damage to accumulate [1]. The DNA damage repair gene* XRCC1* (X-ray repair cross complementing gene 1) functions in base excision repair and the repair of single-strand breaks that are caused by ionizing radiation and oxidative damage [2]. Importantly, polymorphisms in* XRCC1* are correlated with susceptibility to various tumors [3–6].

Esophageal cancer (EC), a common malignancy of the digestive tract, has a complex etiology and is currently believed to result from combined genetic and environmental factors [7]. Several genes, including* XRCC1*, are associated with EC risk [8]. Environmental factors including smoking history, alcohol consumption, and nutrition have also been associated with EC risk [9]. At least one polymorphism in* XRCC1*, Arg194Trp, has been associated with the occurrence of EC; in contrast, the Arg280His variant appears not to be associated with the occurrence of EC, and conflicting reports have made the contribution of the Arg399Gln variant to the disease unclear [10–13]. Further, potential interactions between* XRCC1* variants and environmental factors have not been resolved. Indeed, in a population with a family history of EC, affected individuals with exposure to the same environmental factors account for just a small proportion [14, 15]. This suggests that individual genetic susceptibility to environmental exposures is associated with the occurrence of EC; thus, interaction(s) between genetic susceptibility and environmental factors likely contribute to EC etiology.

To investigate the potential interaction(s) between genetic variation and an environmental factor, alcohol consumption, Korean and Han Chinese individuals with esophageal cancer were selected from the Harbin area of China, which is inhabited by more Korean people. The distribution of the Arg399Gln polymorphism of* XRCC1* was identified, and genotypes were studied in relation to both esophageal cancer and alcohol consumption.

## 2. Participants and Methods

### 2.1. Participants

This prospective study included 203 patients with esophageal cancer (case group), who visited the Harbin Medical University Cancer Hospital between July 2013 and June 2014. Of these, 124 were males, and 79 were females; 115 were Han Chinese and 88 were Korean. Their mean age was 60.7 ± 8.1 years. All patients underwent surgery or gastroscopy and were pathologically confirmed to have esophageal squamous cell carcinoma. Additionally, other possible bodily tumors were ruled out, and patients did not undergo radiotherapy or chemotherapy. For the control group, 210 patients with nondigestive disorders who were hospitalized during the same period were randomly selected. Of them, 113 were males and 97 were females; 105 were Han Chinese and 105 were Korean. The mean age of controls was 58.6 ± 6.5 years. The control and EC groups were not significantly different in age or sex distribution (*P* > 0.05). All participants provided informed consent. This study was approved by the Ethics Committee of the Harbin Medical University Cancer Hospital.

Peripheral blood (5 mL) was collected from each subject into EDTA-coated tubes and stored at −80°C. Alcohol consumption history was collected from both groups using questionnaires. Participants were considered as alcohol consumers if they consumed an alcoholic beverage once per week and of at least 40 mL of alcohol.

### 2.2. Genomic DNA Extraction

DNA extraction from whole blood was performed according to the instructions in the AxyPrep blood genomic DNA kit (Axygen Biosciences, CA, USA), as follows: 5 mL of peripheral blood was anticoagulated with sodium citrate to isolate and lyse white blood cells. Sodium precipitation was used to extract DNA, and extracted DNA was stored at −20°C.

### 2.3. Genotyping

Genotypes for* XRCC1* were determined by PCR-restriction fragment length polymorphism. Primer sequences for* XRCC1* amplification were designed and synthesized by Sangon Biotech (Shanghai, China). The upstream primer for Arg399G1n was 5′-TTGTGCTTTCTCTGTGTCCA-3′, the downstream primer was 5′-TCCTCCAGCCTTTTCTGATA-3′, and the anticipated product was 615 bp. PCR amplification was performed in a PCR Thermal Cycler Dice (TaKaRa Biotech Co. Led, Code TP600, Dalian, China) using the following conditions: predenaturation at 94°C for 5 min; 35 cycles of denaturation at 94°C for 30 s, annealing at 61°C for 30 s, and extension at 72°C for 45 s; and extension at 72°C for 7 min. PCR products were subjected to restriction digestion with* Msp* I (TaKaRa Biotech Co. Led, Dalian, China) overnight at 37°C in a water bath. Five internal positive controls were prepared at the same time. Digested products were separated by agarose gel electrophoresis (2%) and visualized by a gel imaging system.

**Figure 1:**
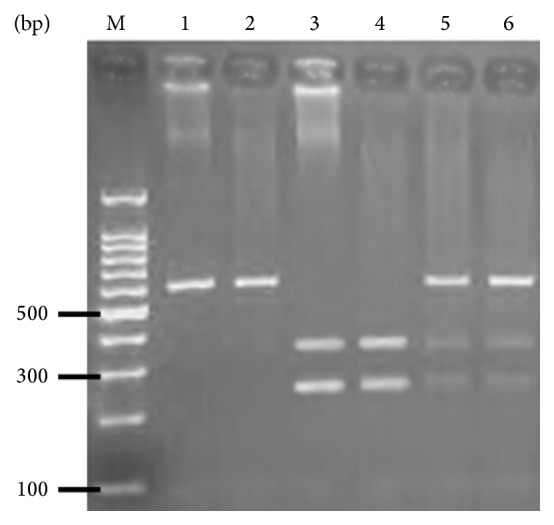
The* XRCC1* Arg399Gln locus was amplified by PCR and PCR products were digested with* Msp* I to detect the 3 possible genotypes.* Lane M*: 100 bp DNA Marker I;* Lanes 1-2*, Gln/Gln;* Lanes 3-4*, Arg/Arg;* Lanes 5-6*, Arg/Gln.

Restriction digestion for Arg399Gln produced the following product sizes: the wild-type genotype (Arg/Arg) was 374 bp and 221 bp; the heterozygous genotype (Arg/Gln) was 615 bp, 374 bp, and 221 bp; and the homozygous variant (Gln/Gln) was 615 bp (Figure 1).

### 2.4. Statistical Treatment

SPSS 17.0 was used to analyze the data. Measurement data are expressed as mean ± standard deviation. A chi-square test was used to compare the allelic and genotypic frequencies in the case group and control group, and the genotype distributions in both groups were compared using a chi-square contingency table. Odds ratios (OR) and 95% confidence intervals (CI) were used to study the correlation of various genotypes and alcohol consumption with the risk of esophageal cancer.

## 3. Results

### 3.1. *XRCC1* Arg399Gln Genotype Distributions in Esophageal Cancer Cases and Controls of Korean and Han Chinese Descent

Hardy-Weinberg equilibrium was used to test the genotype distributions of the* XRCC1* Arg399Gln locus in both Korean and Han Chinese populations. The three possible genotypes were Arg/Arg, Arg/Gln, and Gln/Gln (Figure 1). For each group, the distributions met Hardy-Weinberg equilibrium.

In comparing the EC group with the control group, the allelic and genotypic frequencies at Arg399Gln were not significantly different (*P* > 0.05; Table 1). The gene mutation was considered an exposure and the wild-type genotype was the control; there were no marked differences in the occurrence of EC in subjects with various genotypes. Further, the allelic and genotypic frequencies were compared in individuals with EC of Korean and Han Chinese descent, and there were no significant differences between the groups (*P* > 0.05; Table 2). Additionally, the allelic and genotypic frequencies by nationality were compared between EC cases and controls; no significant difference was detected.

### 3.2. Interaction between Alcohol Consumption and* XRCC1* Arg399Gln Polymorphism on Risk of EC

Within the EC group, participants were stratified by genotype and alcohol consumption history (Table 3). The reference group comprised individuals with the wild-type Arg/Arg genotype who did not consume alcohol. Of those who consumed alcohol, the odds ratios (95% CI) for the Arg/Arg and Arg/Gln genotypes were 3.539 (2.039–6.142; *P* < 0.05) and 0.653 (0.359–1.187; *P* > 0.05), respectively.

## 4. Discussion

Previous research has produced conflicting results regarding the contribution of the* XRCC1* Arg399Gln polymorphism to the risk of esophageal cancer [10–13]. One study demonstrated an increased risk of EC in individuals with the Gln/Gln genotype, and the Arg399Gln polymorphism was correlated with susceptibility to EC [10]. In contrast, our study identified no differences in the genotypic distributions between individuals with EC and healthy controls. This finding supports those of other studies [11, 13]. The difference in results between this/similar studies [11, 13] and conflicting studies [10, 12] may result from variations in sample size or geographic distributions. Additionally, confounding factors like lifestyle or environmental influences were not investigated in all prior studies. No increased likelihood of EC was identified for any genotype, which indicates that the* XRCC1* Arg399Gln polymorphism may not be correlated with EC in Harbin city (Heilongjiang Province, China). In addition, the genotypic distributions did not differ among Korean and Han Chinese nationalities.

Because previous studies did not assess interactions between environmental factors and* XRCC1* genotype on EC risk, alcohol consumption history was investigated as a potential interacting factor here. Interestingly, compared with individuals with EC who had a wild-type genotype and did not consume alcohol, those who consumed alcohol were ~3.5 times less likely to develop EC if they had the wild-type Arg/Arg genotype.

Thus, the findings of this study corroborate previous results indicating that the* XRCC1* Arg399Gln polymorphism does not contribute to EC risk. However, alcohol consumption may be an important interacting factor that decreases EC risk in individuals with the wild-type genotype.

## Figures and Tables

**Table 1:** Analysis of correlation between the *XRCC1* Arg399Gln polymorphism and susceptibility to esophageal cancer (*n* (%)).

Groups	*n*	Arg/Arg	Arg/Gln	Gln/Gln	A	G
Case group	203	102 (50.2)	78 (38.4)	23 (11.3)	282 (69.5)	124 (30.5)
Control group	210	125 (59.5)	63 (30.0)	22 (10.5)	313 (74.5)	107 (25.5)
*χ* ^2^		3.831			2.630	
*P*		0.147			0.105	

**Table 2:** Analysis of correlation between the *XRCC1* Arg399Gln polymorphism and susceptibility to esophageal cancer by nationality (*n* (%)).

EC population	*n*	Arg/Arg	Arg/Gln	Gln/Gln	A	G
Han Chinese	115	55 (47.8)	48 (41.7)	12 (10.4)	148 (67.3)	72 (32.7)
Korean	88	47 (53.4)	30 (34.1)	11 (12.5)	124 (70.5)	52 (29.5)
Total	203	102	78	23	282	124
*χ* ^2^		1.256			0.460	
*P*		0.534			0.498	

**Table 3:** The interaction between alcohol consumption history and *XRCC1* Arg399Gln polymorphism (*n* (%)).

Alcohol consumption history	Genotype	Control group	Case group	OR	95% CI	*χ* ^2^	*P*
Yes	Arg/Arg	88 (68.2)	41 (31.8)	3.539	2.039–6.142	20.886	0.000
	Arg/Gln	39 (48.1)	42 (51.9)	0.653	0.359–1.187	1.961	0.161
	Gln/Gln	14 (46.7)	16 (53.3)	0.693	0.304–1.582	0.761	0.383
	AG + GG	53 (56.1)	58 (43.9)	0.664	0.382–1.154	2.120	0.145

No	Arg/Arg	37 (37.8)	61 (62.2)	Reference			
	Arg/Gln	24 (40.0)	36 (60.0)	0.910	0.471–1.758	0.079	0.778
	Gln/Gln	8 (53.3)	7 (46.7)	0.531	0.178–1.584	1.317	0.251
	AG + GG	30 (47.6)	33 (52.4)	0.667	0.351–1.267	1.536	0.215
